# An impact of type 2 diabetes mellitus on NKT-like cell population in humans: a new insight into impaired immune response in hyperglycemia

**DOI:** 10.3389/fendo.2025.1641318

**Published:** 2025-08-26

**Authors:** Emilia Adamska-Fita, Przemysław Wiktor Śliwka, Bartłomiej Stasiak, Małgorzata Karbownik-Lewińska, Andrzej Lewiński, Magdalena Stasiak

**Affiliations:** ^1^ Department of Endocrinology and Metabolic Diseases, Polish Mother’s Memorial Hospital – Research Institute, Lodz, Poland; ^2^ Department of Endocrinology and Metabolic Diseases, Medical University of Lodz, Lodz, Poland; ^3^ Institute of Information Technology, Lodz University of Technology, Lodz, Poland

**Keywords:** NKT-like cells, CD4-CD8-NKT-like cells, diabetes mellitus, T2DM, glucose, immune response, IFN-γ

## Abstract

**Background:**

Type 2 diabetes mellitus (T2DM) is a chronic metabolic disease characterized by insulin resistance and pancreatic β-cell dysfunction. T2DM is associated with increased risk of infections and of several malignancies, although the underlying immune mechanisms remain not fully elucidated. Natural Killer T-like cells (NKT-like) belong to a unique subpopulation of T lymphocytes defined by expression of the markers specific to NK (Natural Killer) cells (CD56) and T cells (TCR - T cell receptor). As NKT-like cells possess unique cytotoxic properties, they form a bridge between innate and adaptive immunity. The aim of our study was to assess associations between the presence of T2DM and the profile of NKT-like cell subpopulations.

**Methods:**

Peripheral blood mononuclear cells (PBMCs) were obtained from 86 patients. NKT-like cells were subsequently isolated from the PBMC fraction using a CD3+ CD56+ NKT cell isolation kit in combination with magnetic bead-based separation. To evaluate NKT-like cells subpopulations distribution, flow cytometry (FC) was used. NKT-like cells were categorized into CD4-CD8- (double negative, DN), CD4+CD8-, CD4-CD8+, and CD4+CD8+ (double positive, DP) subpopulations, with further subdivision of DP and CD4-CD8+ subpopulations into CD4highCD8mid/CD4midCD8high and CD4-CD8mid/CD4-CD8high subpopulations, respectively. Associations between NKT-like cells subpopulation and T2DM, and – additionally – correlations between NKT-like and glucose levels and body mass index (BMI) were evaluated.

**Results:**

T2DM group demonstrated a significantly diminished percentage of DN NKT-like cells as compared to control group. A strong negative correlation was observed between DN NKT-like cell levels and glucose concentration, but not BMI. Based on further subdivision of DP and CD4-CD8+ subpopulations a significant negative correlation was also observed between glucose levels and the CD4^-^CD8mid NKT-like cell subpopulation. No such association was detected for the other subpopulations.

**Conclusions:**

Our study demonstrated that DN NKT-like cells, which possess significant cytotoxic activity, are depleted in T2DM patients. These results may explain the novel potential mechanism of increasing susceptibility to infections and cancers in T2DM and emphasize the need for precise glycemic control. The novel insights into NKT-like cell immunomodulation role in T2DM may open new, targeted therapies in metabolic diseases. Further research in larger cohorts is needed to confirm these pioneering observations.

## 1 Introduction

Type 2 diabetes mellitus (T2DM) is a chronic metabolic disorder characterized by insulin resistance and progressive pancreatic β-cell dysfunction, leading to hyperglycemia (1). The pathophysiology of T2DM involves a combination of various factors including chronic low-grade inflammation and dysregulation of the immune system (2–4). In 1993, Hotamisligil et al. conducted pioneering research in which a link between inflammation and metabolic dysfunction was found. Authors revealed that tumor necrosis factor alpha (TNF-α), produced by adipose tissue, is a significant mediator of insulin resistance in the context of obesity (5). The underlying mechanisms of T2DM pathogenesis and complications are still incompletely understood, but many studies have proven that immunocompetent cells including T cells, B cells, Natural Killer (NK) cells, and macrophages infiltrate adipose tissue and play a crucial role in adipose inflammation, and in the development of insulin resistance which leads to the onset of T2DM (6–11). However, little is known about the involvement of other immune cells, including Natural Killer T (NKT) cells and Natural Killer T-like (NKT-like) cells, in the pathogenesis of T2DM-related complications.

NKT-like cells, until recently often referred as NKT cells, are a unique, heterogenous subset of T lymphocytes that share properties of both conventional T cells (i.e. T cell receptor - TCR) and NK cells (i.e. cluster of differentiation (CD) 56). Classically, NKT cells were divided into two primary categories: type I NKT cells (invariant NKT - iNKT) and type II NKT cells (variant - vNKT). The classical identification of NKT cells, based on CD3 and CD56 surface antigens, have been reconsidered with the discovery of unspecific expression of CD56 molecule on more broad spectrum of T lymphocytes – mainly activated γδ T lymphocytes and a percentage of αβ T lymphocytes (12). It is, however, strongly considered, that cells expressing CD56 antigen are characterized by higher state of activation, exhibiting some level of cytotoxic properties, therefore, possibly sharing similar properties (13). Unlike conventional T cells that recognize peptide antigens presented by classical major histocompatibility complex (MHC) molecules, iNKT cells recognize lipid antigens presented by the non-classical MHC molecule CD1d (14). Invariant NKT cells are very rare in peripheral blood unlike other subpopulations of NKT-like cells, that occur more frequently in the circulation and their activation is independent of CD1d-mediated antigen presentation. NKT-like cells indicate significant cytotoxic activity and produce proinflammatory cytokines such as IFN-γ and TNF-α, contributing to their anti-tumor and antimicrobial defense (15). NKT-like cells serve as a link between innate and adaptive immunity, exhibiting cytotoxic capabilities through the release of perforin and granzyme B and producing a diverse array of cytokines that modulate inflammatory responses both directly, and indirectly by interacting with various immune cells (16). Recent advances in immunology, particularly in cell identification techniques, have led to ongoing revisions in the classification of iNKT and NKT-like cell subpopulations. Depending on CD4 and CD8 expression, iNKT and NKT-like cells can be categorized into four subpopulations: CD4-CD8+, CD4+CD8, CD4+CD8+ (double positive; DP) and CD4-CD8- (double negative; DN) (17). In the past, CD4-CD8- and CD4-CD8+ NKT cells were considered a part of the same subpopulation (18, 19). However, recent research has demonstrated significant gene expression differences between DN and CD4-CD8+ NKT cells, highlighting their distinct identities (19).

NKT-like cells as extraordinary subset of T lymphocytes characterized by potent cytotoxic activity and their ability to respond rapidly through cytokine production have regulatory effects which makes them a target for new therapeutic interventions related to infections, cancer, and autoimmune disorders (15, 20–23).

In the recent years, significant advancement in immunology is observed, particularly in the characterization of immune cell subpopulations. The application of high-dimensional cytometry, single-cell RNA sequencing, and detailed profiling of surface and functional markers has facilitated the identification of novel, functionally distinct subpopulations of immune cells. Data on the impact of T2DM on NKT-like cells are scarce and include only the influence on a total number of NKT-like cells (15, 24, 25). There is a lack of comprehensive study on the impact of NKT-like cell subpopulations on T2DM-related complications. Therefore, the aim of the present study was to evaluate potential association between the presence of T2DM the distribution of CD4/CD8-defined subpopulations within NKT-like cells, as well as correlation between glucose concentration and the distribution of NKT-like subpopulations, in order to find potential mechanisms which can lead to impaired immune response in T2DM via modification of NKT-like cell subpopulation profile.

## 2 Materials and methods

### 2.1 Patients

The research included 86 patients (66 women and 20 men) treated at the Department of Endocrinology and Metabolic Diseases of the Polish Mother’s Memorial Hospital – Research Institute in Łódź, Poland. The study group included 24 patients with T2DM (DM group, DMG) and 62 persons without glucose metabolism-related disorders (control group, CG). Individuals with prediabetic states, active malignancy, active infection or inflammatory diseases which might have influenced the results were excluded from the study.

### 2.2 Biochemical analysis

Glucose concentration was assessed using electrochemiluminescence immunoassay (ECLIA) method on the Cobas e601 analyzer (Roche Diagnostics, Indianapolis, IN, USA).

### 2.3 NKT-like cell isolation

Peripheral blood samples (2 × 4.9 mL) were obtained from each participant into EDTA-coated collection tubes (Sarstedt, Nümbrecht, Germany) using standard venipuncture techniques. Peripheral blood mononuclear cells (PBMCs) were subsequently isolated via density gradient centrifugation with Histopaque^®^-1077 (Thermo Fisher Scientific, Waltham, MA, USA), performed at 400 × g for 30 minutes at room temperature. In order to isolate NKT-like cells, the PBMCs fraction was subjected to two-step magnetic separation using a CD3+ CD56+ NKT Cell Isolation Kit in combination with a magnetic column system (Miltenyi Biotec, Bergisch Gladbach, Germany). Cell purity was assessed individually for each subject, yielding a median purity of 93.2% across samples, with values ranging from 80.1% to 99.1%. Downstream analyses were conducted on cells gated as CD3+CD56+ to ensure population specificity.

### 2.4 Flow cytometry

In order to examine the distribution of NKT-like cell subsets, flow cytometry (FC) was employed. Cells were stained with fluorochrome-conjugated monoclonal antibodies specific to human CD3 (APC, clone UCHT1) and CD56 (PE-Cy7, clone B159), (both Becton Dickinson, Franklin Lakes, NJ, USA) for NKT-like cell identification. Anti Vα24JαQ TCR Chain antibody (PE, clone 6B11) was used to determine the percentage of Invariant NK T Cell population resulting in very low (~1.2%) frequency of this cell subtype. We did not analyze the αβ T-cell receptor nor the γδ T-cell receptor on the surface of isolated cells, therefore, according to current standards, we assume that all further subset analysis are performed on heterogenous population of CD3+CD56+ NKT-like cells. Subset differentiation was further refined using antibodies targeting CD4 (FITC, clone SK3) and CD8 (PerCP, clone SK1) surface markers (both Becton Dickinson, NJ, USA). Isotype-matched control antibodies were included in each experiment to verify staining specificity. Flow cytometric analysis was performed on a BD FACSCanto II cytometer (Becton Dickinson, NJ, USA), and data were analyzed using the associated software.

**Figure 1:**
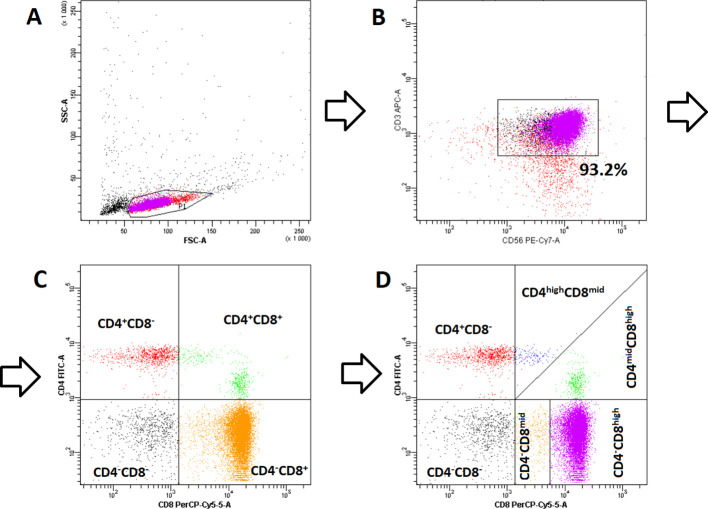
Representative flow cytometry (FC) plots illustrating the gating strategy used for cell analysis: **(A)** PBMC-derived population post-magnetic enrichment; **(B)** CD3^+^CD56^+^ NKT-like cells following FC purification; **(C)** Primary gating scheme for initial NKT-like cell identification; **(D)** Refined gating strategy for distinguishing NKT-like cell subpopulations.

Based on the framework established by Montoya et al. (14) for invariant NKT cells (iNKT), four primary subsets were initially defined: CD4-CD8- (double negative), CD4-CD8+, CD4+CD8-, and CD4+CD8+ (double positive). Subsequent high-resolution analysis revealed notable heterogeneity within the population of cells expressing CD8 molecule. As a result, CD4+CD8+ cells were further stratified into CD4highCD8mid and CD4midCD8high subtypes, while CD4-CD8+ cells were subdivided into CD4-CD8mid and CD4-CD8high subtypes. The detailed gating strategy used for these analyses is shown in **Figure 1**.

### 2.5 Statistical analysis

For each patient, the number of NKT-like cells of each type (CD4-CD8-, CD4-CD8+, CD4+CD8-, CD4+CD8+) was obtained, as detailed above, and expressed as a percentage of the total number of NKT-like cells gated as CD3+CD56+ cells. Additionally, the number of CD4highCD8mid cells (and – similarly – of CD4midCD8high cells) was also expressed as a percentage value. In this case, we used two different representations: percentage with respect to the total number of NKT-like cells (as above) and percentage with respect to the number of CD4+CD8+ cells only (note that in this representation CD4highCD8mid sums up to 100% with CD4midCD8high). Similarly, the numbers of CD4-CD8mid and of CD4-CD8high cells were also transformed to percentages in the same two ways: with respect to the total number of NKT-like cells and with respect to the number of CD4-CD8+ cells only. All the analyses reported in the present study were based on thus defined percentage values (no absolute values of NKT-like cell counts were used).

The NKT-like cell percentages were subjected to statistical analysis including empirical distribution assessment of each NKT-like type/subtype within the DM and control groups. In both groups, summary statistics (mean, standard deviation and order statistics) were computed for all NKT-like types/subtypes and statistical tests were performed to reveal statistically significant differences between DMG and CG. Student’s t-test was used for normally distributed variables and Mann-Whitney U-test otherwise. The normality of distribution was assessed with Shapiro-Wilk test. Additionally, for contingency tables obtained from splitting the patients into two groups on the basis of NKT-like cell percentage, chi-squared test was applied. In the case of real-valued variables (glucose level and body mass index) Pearson’s linear correlation coefficient and Spearman’s rank correlation coefficient with respect to NKT-like cell percentage were computed. All the computations were performed with the use of SciPy library for Python programming language.

### 2.6 Ethics procedures

All patients provided written informed consent for the procedures after receiving a full explanation of their purpose and course. The study received approval from the Ethics Committee of the Polish Mother’s Memorial Hospital – Research Institute in Łódź, Poland (approval code: 41/2021).

## 3 Results

The study group included 24 patients with T2DM and 62 persons without glucose metabolism-related disorders. The mean age of the included patients was 59.7 ± 13.7 years. The mean age of patients in DMG and CG was 64,96 ± 11,3 and 58,25 ± 14,1 years, respectively. The whole cohort included 66 females and 20 males, and the proportion of males to females in DMG and CG was 9:15 and 11:51 respectively. The mean level of glucose in DMG was 125.88 mg/dl, median 122.5 mg/dl. The mean level of glucose in CG was 91.08 mg/dl, median 90.0mg/dl.

Taking into account the differences between DMG and CG with regard to gender, with significantly higher proportion of males in DMG, we performed an initial analysis of potential association between gender and NKT-like cell subpopulation. No association between gender and the percentage of NKT-like cells was found, with *p* value of 0.866, 0.826, 0.388 and 0.203 for CD4+CD8-, CD4+CD8+, CD4-, CD8+ and CD4-CD8- subpopulations, respectively, which confirmed the lack of gender-related bias.

In the first part of the main study, we analyzed a possible relationship between our main four NKT-like cell subpopulations and the presence of T2DM. Student t-test and Mann-Whitney test were used to compare the percentage of NKT-like cells (of a given subpopulation) between DMG and CG. This analysis showed significant negative association (*p*-value = 0.007) between the presence of T2DM and the percentage of CD4-CD8- NKT-like cells (with respect to the total number of NKT-like cells gated as CD3+CD56+ cells, as described above). In the case of the other NKT-like cell types, no significant association with T2DM was found. **Table 1** presents the obtained results (only Mann-Whitney test results are reported, as most of the considered distributions deviated significantly from the normal distribution.

**Table 1:** Associations between percentage of NKT-like cell types and T2DM.

		mean ± SD	Median	Min	max	IQR	*p*-value
CD4+CD8-	CG	17.61 ± 15.57	13.4	0.3	62.1	19.7	0.647
CD4+CD8-	DMG	17.08 ± 16.63	10.2	0.6	65.9	22.78	
CD4+CD8+	CG	6.93 ± 9.94	3.25	0.5	52.3	5.68	0.394
CD4+CD8+	DMG	8.23 ± 10.68	5.8	0.3	50.0	7.45	
CD4-CD8-	CG	16.67 ± 15.84	10.3	0.0	60.7	14.6	**0.007**
CD4-CD8-	DMG	8.28 ± 8.76	6.4	0.4	36.0	7.33	
CD4-CD8+	CG	58.81 ± 20.39	62.1	11.2	91.6	28.93	0.112
CD4-CD8+	DMG	66.41 ± 24.36	68.45	7.7	97.3	36.28	

Statistically significant p-values are presented in bold.

We explored further the dependence between CD4-CD8- and T2DM, by splitting all the patients into two subgroups on the basis of the number of CD4-CD8- cells. We set the percentage threshold to 9%, as this particular value yielded approximately equal-sized groups: 44 patients below the threshold (i.e. having less than 9% of CD4-CD8- cells among all four studied NKT-like cell types gated as CD3+CD56+ cells) and 42 patients over the threshold (with more than 9% of CD4-CD8- cells). The corresponding contingency is presented in **Table 2**. We performed chi-squared test which clearly confirmed the correlation with T2DM (χ^2^ = 5.16, *p*-value = 0.023). The obtained odds ratio (OR=0.318) indicates that the incidence of T2DM in the group with high CD4-CD8- percentage (7 vs 35) is over three times lower than in the control group (17 vs 27). It should be stressed that these results are stable in a range of threshold values (with the maximum at threshold of 10% which generates groups of 47 and 39 patients respectively, yielding χ^2^ = 5.56, *p*-value = 0.018 and OR=0.293).

**Table 2:** Contingency table for two binary variables: CD4-CD8- (after thresholding) and DM.

	No diabetes	Diabetes
Percentage of CD4-CD8- < 9%	27	17
Percentage of CD4-CD8- ≥ 9%	35	7

**Figure 2:**
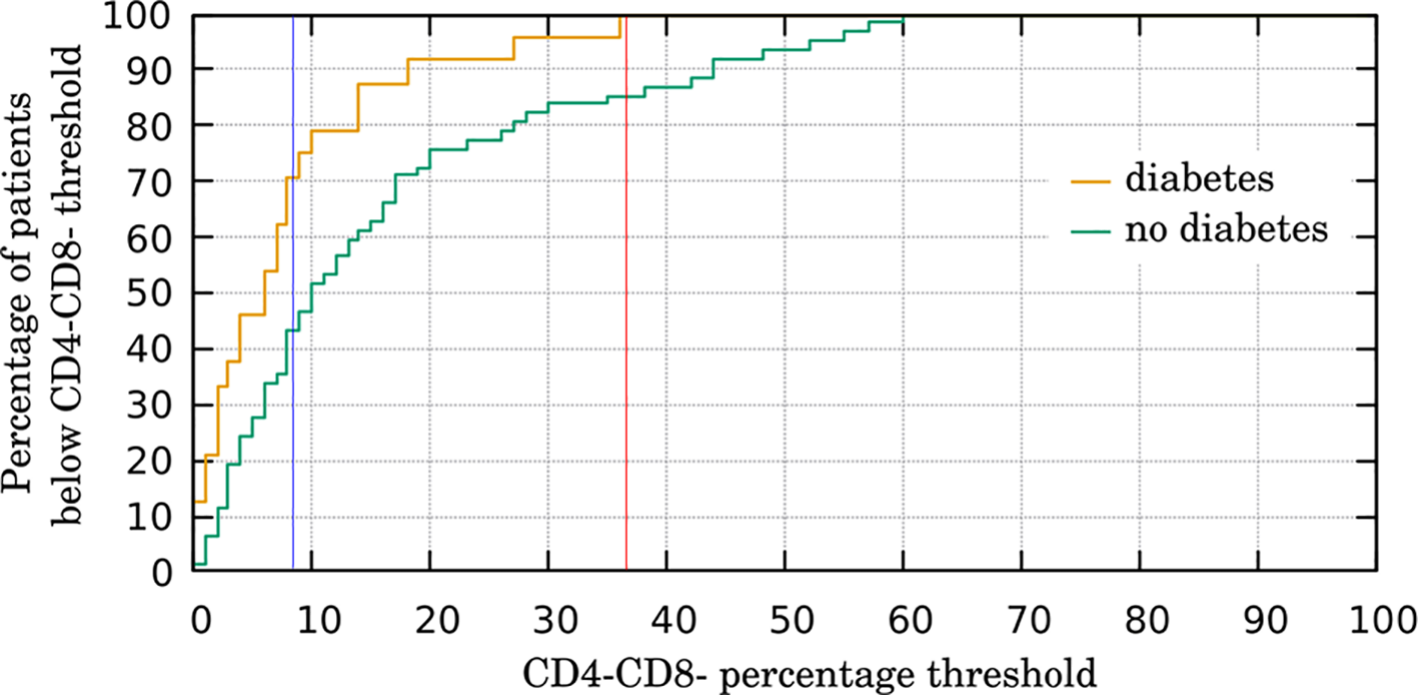
The proportion of patients with low CD4-CD8- percentage in peripheral blood in type 2 diabetes group (DMG) (n = 24) and control group (CG) (n = 62). Yellow plot presents how many DMG patients (among all DMG patients) have CD4-CD8- percentage (with respect to all studied NKT-like cell types) lower than a given threshold. Green plot presents how many CG patients (among all CG patients) have CD4-CD8- percentage (with respect to all studied NKT-like cell types) lower than a given threshold. Blue vertical line presents a specific case (approximately equal-sized groups) described in the text and in **Table 2**; chi-squared test value: 5.16, p-value: 0.023. Red vertical line presents a specific case for the threshold of 37% (all DMG patients have the CD4-CD8- percentage below this threshold) described in the text; chi-squared test value: 3.891, p-value: 0.049).

**Figure 2** shows how many T2DM patients (yellow line) and CG individuals (green line) fall below the threshold, for all possible threshold values. To make comparison more clear, we present the percentage instead of absolute numbers (i.e. the number of T2DM patients below the threshold with respect to all T2DM patients – and similarly for the CG persons). The curves obtained in this way may be interpreted as the estimates of cumulative distribution function (CDF) of CD4-CD8- level in DMG and CG, respectively. For example, all T2DM patients have CD4-CD8- below 37%, so the yellow line hits 100% for the threshold of 37%, while the green line reaches only 85% at this point, as 9 non-DM patients (out of 62) have CD4-CD8- level higher than 37%. It is worth noting that the *p*-value for this particular case is 0.049.

**Figure 3:**
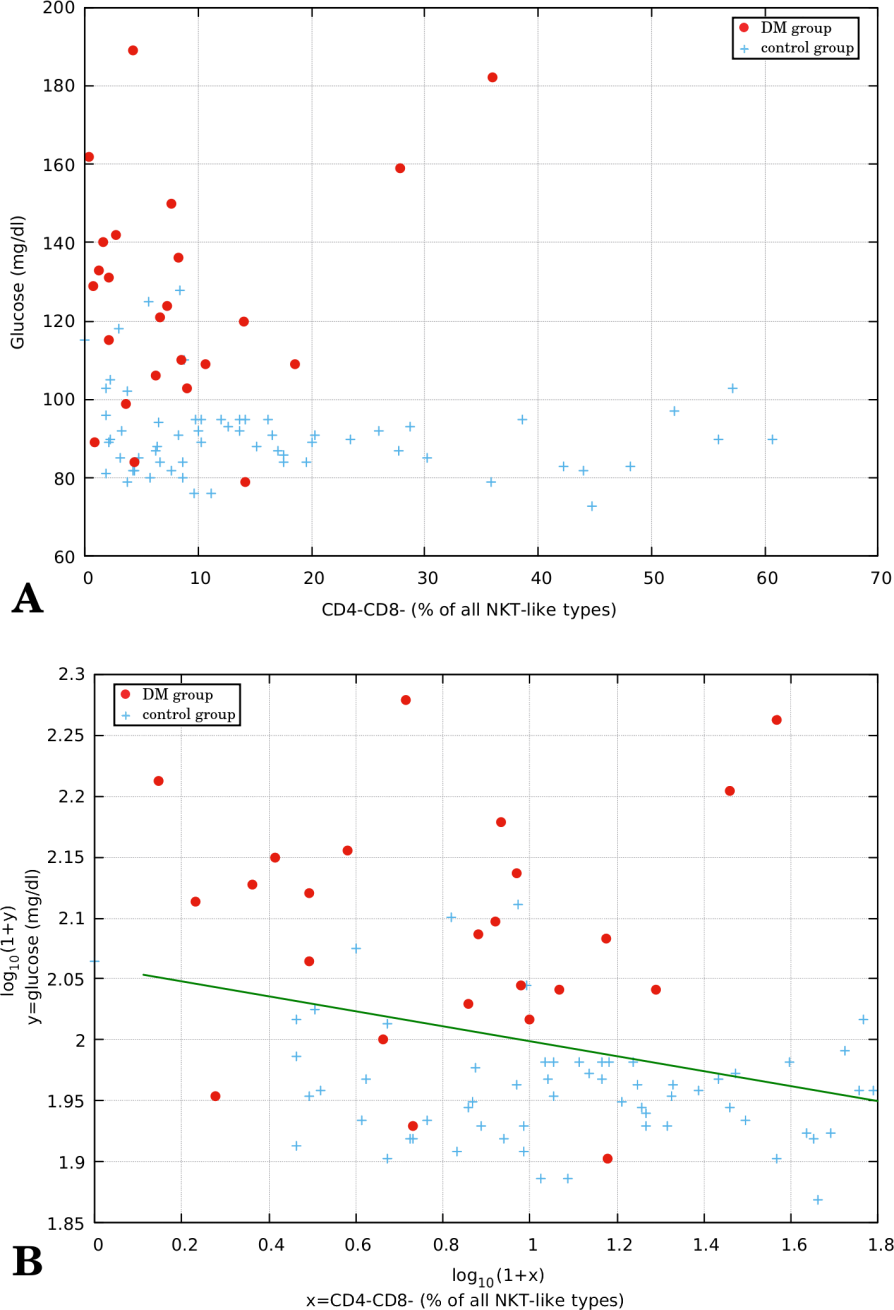
**(A)** Relation between glucose level (mg/dl) and CD4-CD8- percentage in peripheral blood of type 2 diabetes group (n = 24) and control group (n = 62). **(B)** The same relation after logarithmic scaling of both axes. The linear regression parameters are -0.062 and 2.06 (slope and intercept, respectively) and Pearson’s *ρ:* -0.29 with *p*-value: 0.007 (plot B). Spearman’s *ρ*: -0.27 with *p*-value: 0.011 (both plots: A and B).

In the further analysis we evaluated correlation between CD4-CD8- percentage (with respect to the total number of NKT-like cells) and glucose level. **Figure 3A** shows the scatter plot of glucose level vs CD4-CD8- percentage in our whole study group (DMG + CG). The negative trend is clearly visible here although it is non-linear so we decided to use Spearman’s rank correlation coefficient (which yields *ρ* = -0.27 with *p*-value 0.011) instead of Pearson’s *ρ*. Linear correlation is better visible after logarithmic scaling of both variables (**Figure 3B**). Pearson’s correlation coefficient after this transformation is equal to -0.29 with *p*-value = 0.007 (Spearman’s *ρ* remains equal to -0.27, due to the rank-preserving monotonicity of the log function).

**Figure 4:**
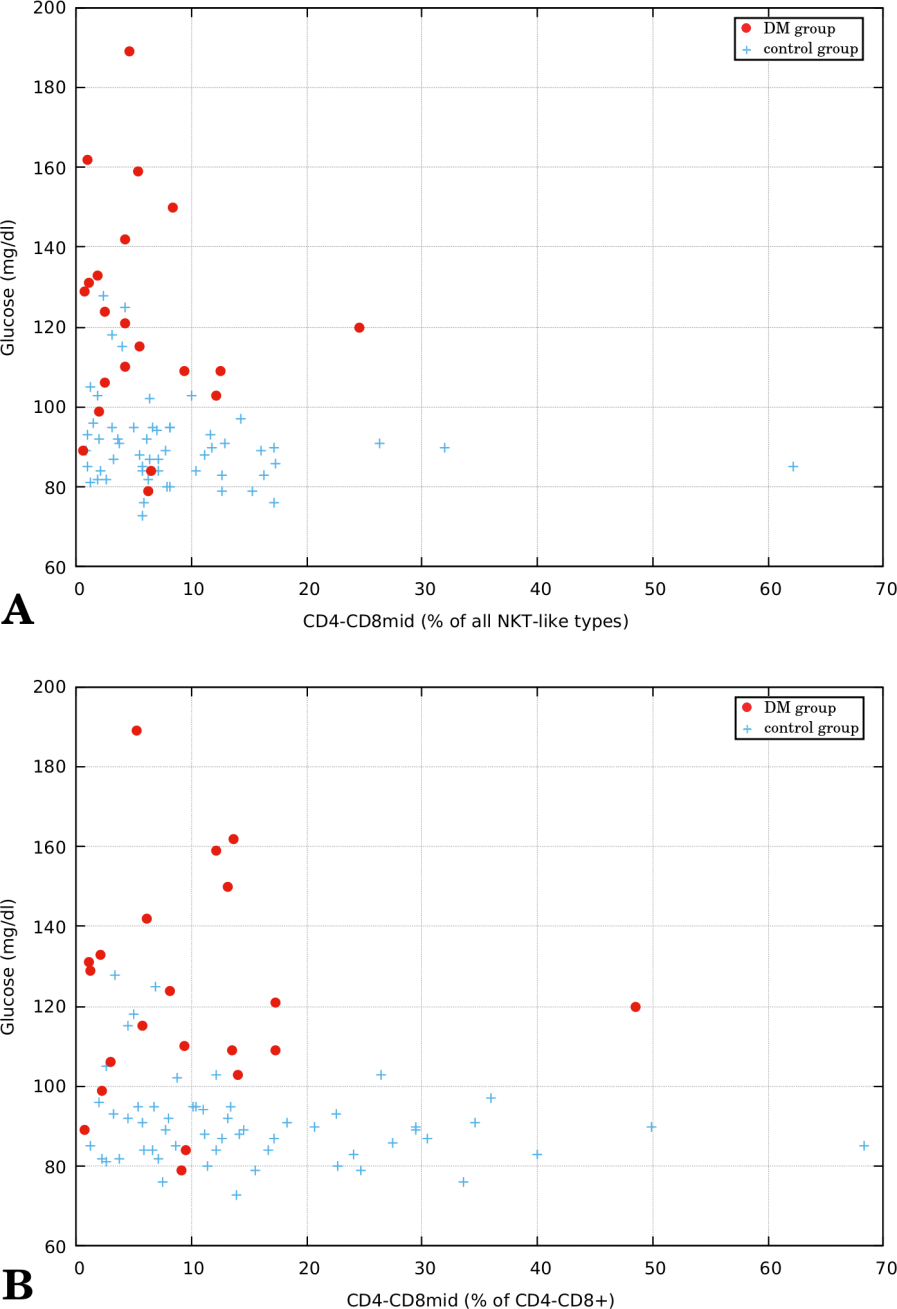
Relation between glucose level (mg/dl) and CD4-CD8mid percentage in peripheral blood of type 2 diabetes group (DMG, n = 21) and control group (n = 56). **(A)** CD4-CD8mid percentage represented as % of all studied NKT-like cell types (Spearman’s *ρ:* -0.292 with *p*-value: 0.01). **(B)** CD4-CD8mid percentage represented as % of CD4-CD8+ cells (Spearman’s *ρ*: -0.25 with *p*-value: 0.029).

In the next part of the study, we additionally analyzed correlation of the subtypes of NKT-like cells with glucose level. The analysis revealed negative correlation between glucose and CD4-CD8mid subtype. This correlation was visible irrespective of the method of CD4-CD8mid level representation – either as percentage of all four studied NKT-like types or as percentage of CD4-CD8+ cells only (see Section 2.5). The first case is depicted in **Figure 4A** (Spearman’s *ρ* = -0.292 with *p*-value = 0.01) and the second one – in **Figure 4B** (*ρ* = -0.25 with *p*-value 0.029).

The results for all NKT-like subtypes are presented in **Table 3**. It should be noted that the results presented in its lower half are obtained for CD4-CD8mid and for CD4-CD8high cells represented as a percentage of CD4-CD8+ cells, as described in Section 2.5. That is why these two results are basically the same (the only difference is the sign of the correlation coefficient), as CD4-CD8mid is simply equal to (100% - CD4-CD8high) in this case. Similarly, CD4highCD8mid and CD4midCD8high are expressed relative to CD4+CD8+ cells in the lower part of **Table 3** and therefore their results are also symmetrical (and non-significant, with *p*-value = 0.554).

**Table 3:** Correlations between percentage of NKT-like cell subtypes and glucose level.

NKT-like cell subtype (% of all NKT-like cells)	Spearman’s *ρ*	*p*-value
CD4highCD8mid	0.056	0.627
CD4midCD8high	0.209	0.07
CD4-CD8mid	-0.292	**0.01**
CD4-CD8high	0.096	0.407
NKT-like cell subtype (% of CD4+CD8+)		
CD4highCD8mid	-0.068	0.554
CD4midCD8high	0.068	0.554
NKT-like cell subtype (% of CD4-CD8+)		
CD4-CD8mid	-0,249	**0,029**
CD4-CD8high	0,249	**0,029**

Statistically significant p-values are presented in bold.

In the last step of the study, in order to exclude the potential impact of obesity, we evaluated correlation of NKT-like types with BMI and we did not find any significant correlation (**Table 4**). Similarly, for NKT-like subtypes no correlation with BMI was found (all *p*-values for Spearman’s correlation coefficient > 0.326).

**Table 4:** Correlation between percentage of NKT-like cell types and body mass index (BMI).

NKT-like cell type	Spearman’s *ρ* (*p*-value)	Pearson’s *ρ* (*p*-value)
CD4+CD8-	0.18 (0.101)	0.14 (0.187)
CD4+CD8+	0.14 (0.197)	0.11 (0.316)
CD4-CD8-	-0.05 (0.681)	-0.05 (0.666)
CD4-CD8+	-0.1 (0.352)	-0.124 (0.256)

## 4 Discussion

The pathogenesis and complications of T2DM are not only related to metabolic disorders, but there is also increasing evidence for the important role of immune system impairment. Studies analyzing the role of NKT-like cells in T2DM etiology and in the development of complications have remained deficient. The currently available literature focused on this issue is scarce and mainly regarded the absolute counts of circulating NKT-like cells (15, 24, 25) or overall NKT cell population (26, 27), with no analysis of NKT-like types with regard to the CD4 and CD8 molecule expression.

To the best of our knowledge, this study is the first one to assess specific NKT-like cell subpopulations in the peripheral blood of individuals with T2DM in comparison to individuals without glucose-metabolism-related disorders. Our results demonstrated that patients with T2DM had significantly lower percentage of DN NKT-like cells than healthy individuals. In order to further confirm this observation, we searched for a threshold of percentage of DN NKT-like cells which would divide the whole study cohort into two subgroups of approximately equal number of patients. This analysis demonstrated that the proportion of T2DM to healthy subjects in the group with high DN cells percentage was over three times lower than in the group with low DN cells percentage, and that the group with low DN cells percentage included nearly 2.5 times more patients with T2DM than CG. These results suggested that a distribution of NKT-like cell population was significantly different in T2DM as compared with CG and was associated with lower CD4-CD8- percentage.

The next part of the present study aimed to evaluate correlation between the proportion of NKT-like cell types and glucose level as well as BMI, in order to find out whether the observation of decreased percentage of DN NKT-like cells in T2DM should be considered as a direct consequence of hyperglycemia or as an indirect consequence of immune disorders in obesity. The significant negative correlation was confirmed between DN NKT-like cells and glucose level, while no correlation between any of NKT-like cell types and BMI was found.

Having demonstrated that our results are BMI-independent and related to glucose level, we made a hypothesis that hyperglycemia leads to changes in NKT-like cell population towards cells with no or low CD8 expression. In order to investigate this issue, we additionally analyzed correlation of the distribution of subpopulations of NKT-like cells with glucose level. In our study, NKT-like cells were initially subdivided into four main subpopulations: CD4-CD8-, CD4-CD8+, CD4+CD8- and CD4+CD8+. However, advanced research provided a discovery of significant heterogeneity of both CD8+ subpopulations, which led to further subdivision of CD4+CD8+ and CD4-CD8+ subpopulations into CD4highCD8mid/CD4midCD8high and CD4-CD8mid/CD4-CD8high subtypes. To the best of our knowledge, such subclassification of NKT-like cell subpopulations has never been described in the literature. It was used for subclassification of classic CD8 T cells, however, we would like to underline, that our strict gating strategy and high cell isolation purity let us analyze virtually only the cells expressing CD56. Therefore, CD8+ NKT-like cells analyzed in our study cannot be directly compared to total circulating classic CD8+ T cells.

Having applied this subclassification for NKT-like cells, we demonstrated significant negative correlation between glucose level and the percentage of CD4-CD8mid subtype, while no correlation with other subpopulations was found. This observation confirmed our hypothesis that hyperglycemia can be associated with decreased percentage of NKT-like cells both with no expression or low expression of CD8 molecule. No such correlation was found for CD4-CD8high cells if their percentage with respect to the total number of NKT-like cells was analyzed.

These findings cannot be directly compared to those of other authors due to the absence of similar analyses in the existing studies. However, previous research made attempts to analyze changes of the number of NKT cells and NKT-like cells in T2DM. In the study by Tang et al. the authors found that the frequency and absolute counts of circulating NKT-like cells were significantly lower in patients with T2DM compared to healthy volunteers (15). The study differed significantly from ours, as it included T2DM patients with chronic severe hyperglycemia with a mean HbA1c level exceeding 10%, which indicated a potential presence of multiple T2DM-related complications. In our study, the mean glucose level in T2DM group was 125.88 mg/dl, while in the quoted study it had to be approximately 250 mg/dl (on the basis of HbA1c value). Therefore, our results may be different from those by Tang et al. as very high glucose levels could significantly influence immune responses. We demonstrated that DN NKT-like cell population was depleted, but we did not find a decrease in total number of NKT-like cells. Our study demonstrated that even slight hyperglycemia is associated with impairment of NKT-like cell distribution, and that DN NKT-like cells can be the first or maybe the only population of NKT-like cells depleted in T2DM. In the study by Dworacka et al. (24), no significant difference in the total number of NKT-like cells in the peripheral blood of patients with T2DM compared to healthy controls was found. These results were consistent with the findings by Guo et al. (26) and by Phoksawat et al. (25). Although Guo et al. demonstrated no difference in the total NKT cell number, they observed high level of activated NKT cells in patients with new onset of T2DM and concluded that activated NKT cells played a role in T2DM pathogenesis. Contrary, Dworacka et al. observed that patients with prediabetes presented significantly higher amount of activated NKT-like cells in peripheral blood as compared to both T2DM patients and healthy controls. Moreover, the increased NKT-like cell count in prediabetes was negatively associated with glycated hemoglobin (HbA1c) levels. Authors hypothesized that the diminished number of NKT-like cells in T2DM patients is possibly linked to chronic inflammation and NKT-like cells relocation to vessel walls. On the other hand, Phoksawat et al. (25) did not find any difference in the number of activated NKT-like cells in the peripheral blood of T2DM patients compared to healthy controls. The discrepancies between the studies may be related to a relatively small study groups (24–26) However, the widely observed lack of differences in the total number of NKT-like cells between T2DM and controls may be related to the fact that the changes concern the profile of NKT-like cell subpopulations, as it was demonstrated in our present study, but the total number of cells is not significantly disturbed. In a study conducted by Gajovic et al. (27), oxidative stress caused by hyperglycemia led to a depletion in the amount of splenic NKT cells. Additionally, there was an increase of NKT cells excreting transforming growth factor beta (TGF-β), interleukin (IL)-4, and IL-5. These results led to a hypothesis that T2DM induces a change in cytokine secretion by NKT cells towards a T-helper type 2 (Th2) cytokine profile, which may lead to increased susceptibility of diabetic patients to infections and tumors (27). In this regard, our observations are highly consistent with findings by Gajovic et al., as we demonstrated that T2DM is associated with a decreased percentage of DN NKT-like cells which possess extraordinary cytotoxic properties. Important role in antimicrobial response is connected with innate immunity and production of antimicrobial peptides and cytokines such as interferon-gamma (IFN-γ), tumor necrosis factor alpha (TNF-α) by NKT-like cells (28–30). It was also proven that IFN-γ plays a crucial role in bacterial clearance including *L. monocytogenes* and *M. tuberculosis* infections (31–33). Furthermore, TNF-α, IFN- γ and granzyme B produced by NKT-like cells is also involved in antitumor defense (21, 34–36). In the context of the results of the present study, it seems highly important that the cytokine profile of DN NKT-like cells, particularly their production of IFN-γ, TNF-α is essential for their antitumor and antimicrobial activity (21, 28–36). It should be underlined that the association between T2DM and increased risk of cancer was demonstrated for a number of malignancies, including breast, liver, pancreas, colon/rectum, endometrium, and bladder cancer (37, 38). On the other hand, anti-tumor activity of NKT-like cells was proved (21, 34, 35, 39).

The immunoregulatory function of NKT-like cells in metabolic diseases is further supported by research on type 1 diabetes mellitus (T1DM), which was proven to be associated with abnormalities in NKT-like cells and NKT-cells distribution and function (40–43). Among metabolic diseases, obesity seems to became more and more important, especially because it constitutes the main risk factor of T2DM. Although we did not find any association between the distribution of NKT-like cell populations, other authors demonstrated that pathogenesis of obesity is closely linked to the dysregulation of immune system cells, including NKT-like cells In study conducted by Donninelli et al., accumulation of NKT-like cells was observed in the visceral adipose tissue of obese patients (44). This study does not contradict our results, as tissue distribution and function of NKT-like cells may differ. It was previously demonstrated for NKT cells that they can reduce inflammation in adipose tissue, whereas in the liver, their activation may exacerbate it (45, 46). Future studies analyzing adipose-resident NKT-like subpopulations could provide valuable insights into their role in obesity.

Until very recently, most studies analyzing NKT cells focused either on very unique invariant NKT (iNKT) cells based on Vα24-Jα18 TCR antigen, or on broad spectrum of CD3+CD56+ NKT lymphocytes. When discussing any data, obtained from analysis of NKT-like cells, it should be underlined, that raw comparison of results between different studies is still valid, as long as the same method of NKT-like cells identification was used. We acknowledge, however, that new insights into the heterogeneity of CD3+CD56+ NKT-like cells complicates the discussion of results obtained in various studies, including our study on T2DM patients. We are aware, that the differences in the percentage of any NKT-like cells subtype, based on CD4 and CD8 segregation, might be the reflection of any alternations in cell structure. That includes, but is not limited to: changes in only one NKT-like cell subtype – e.g. αβ T-cells vs. γδ T-cells; different state of activation of various subtypes – including changes in CD56 expression, resulting in different cells being sorted as CD3+CD56+ NKT-like cells; or changes affecting every CD3+CD56+ expressing cell. In the aspect of interpretation of our findings in context of T2DM patients, it is important to mention, that cells expressing CD56 antigen are considered to be activated cells exhibiting some level of cytotoxic properties, therefore, possibly sharing similar properties. Statistical significant differences, between healthy individuals and T2DM patients, may indicate their contribution in the disease mechanisms. Further distinguishing NKT-like cells based on CD4/CD8 markers can provide some initial insights into the subtypes of those cells, pointing on potential subpopulations that are more important for the understanding the immunology of T2DM and helping for the design of further studies in the field.

Taking into account all these observations, the results of our study may constitute an important step in broadening the knowledge on harmful effects of hyperglycemia on immune cells and on mechanisms contributing to the impaired immune defense in T2DM, mainly with regard to increased risk of bacterial infections and complications of infections, as well as of cancer development. The observed depletion represents a novel insight into immune dysregulation in metabolic disease and may serve as a potential therapeutic target. Importantly, this progress would not be possible without detailed analysis of NKT-like subpopulation heterogeneity. The data that NKT-like subpopulations heterogeneity correlate with glucose level suggest that the immune cells subpopulations, rather than total cell counts, should be the core of future studies.

Considering the novelty of our results, the limitations of this study should be taken into account. The main limitation is a relatively small sample size, although it should be noted that no larger cohort has been analyzed with regard to association of NKT-like cell subpopulations and T2DM. Additionally, the DMG included patients treated with different antidiabetic medications, which may influence the results. Moreover, we have not analyzed HbA1c level in our patients and an analysis of correlation of HbA1c and NKT-like cell subpopulations may provide further evidence supporting the results. Our study also did not involve patients with uncontrolled T2DM and extremely high glucose concentrations. Therefore, no conclusion on the impact of severe hyperglycemia on NKT-like cells can be stated. On the other hand, we believe that the strength of this study is the fact that all T2DM patients had similar disease control with only moderately increased glucose level. This fact indicates that even moderate hyperglycemia is associated with changes in NKT-like cell profile with significant decrease in DN subpopulations, which can potentially lead to increased susceptibility to infections as well as increased risk of cancer development. Another strength of our study is the fact that the glucose and NKT-like cell subpopulations were analyzed from the blood collected at the same time point in the same conditions, in order to avoid the impact of other factors, such as meal, physical effort or others.

## 5 Conclusions

Our study has provided evidence that DN NKT-like cell population is decreased in T2DM, and that this phenomenon is related to increased glucose level but not to BMI. As DN NKT-like cells play important role in antimicrobial and antitumor immune defense, the decreased percentage of these cells may explain one of the mechanisms of increased susceptibility and worse course of infections in hyperglycemic conditions as well as increased malignancy risk in T2DM. It is worth emphasizing, that our study revealed that significant depletion of DN NKT-like cells occurs even if glucose level is only moderately elevated. Therefore, our study provides a new point for strict control of glycemia in T2DM. Taking into account the novelty of our findings and the fact that no other research on associations between NKT-like cell subpopulations has been performed so far, our observations should be considered pioneering and they require further confirmation in larger cohorts. These results shed new light on significance of NKT-like cell subpopulations in T2DM, which – if confirmed – may pioneer the way for new therapeutic strategies targeting immune dysfunction in metabolic disorders.

## Data Availability

The raw data supporting the conclusions of this article will be made available by the authors, without undue reservation.
